# Improved survival of mesenchymal stem cells by macrophage migration inhibitory factor

**DOI:** 10.1007/s11010-015-2361-y

**Published:** 2015-02-21

**Authors:** Wenzheng Xia, Congying Xie, Miaomiao Jiang, Meng Hou

**Affiliations:** 1 Department of Neurosurgery, First Affiliated Hospital, Wenzhou Medical University, Wenzhou, China; 2 Department of Radiation Oncology, First Affiliated Hospital, Wenzhou Medical University, Wenzhou, 325000 China; 3 Department of Cardiology, The Second Affiliated Hospital, Harbin Medical University, Harbin, Heilongjiang China

**Keywords:** Mesenchymal stem cells, Hypoxia/serum deprivation-induced apoptosis, MIF, PI3K/Akt-FOXO3a signaling pathways, Oxidative stress

## Abstract

Macrophage migration inhibitory factor (MIF) is a critical inflammatory cytokine that was recently associated with progenitor cell survival and potently inhibits apoptosis. We examined the protective effect of MIF on hypoxia/serum deprivation (SD)-induced apoptosis of mesenchymal stem cells (MSCs), as well as the possible mechanisms. MSCs were obtained from rat bone marrow and cultured in vitro. Apoptosis was induced by culturing MSCs under hypoxia/SD conditions for up to 24 h and assessed by flow cytometry. Expression levels of c-Met, Akt, and FOXO3a were detected by Western blotting. CD74 expression was detected by qRT-PCR, Western blot, and immunofluorescence. Oxidative stress under hypoxia/SD was examined by detection of reactive oxygen species (ROS) and activity of superoxide dismutase (SOD) and malondialdehyde (MDA). Hypoxia/SD-induced apoptosis was significantly attenuated by recombinant rat MIF in a concentration-dependent manner. MIF induced CD74-asssociated c-Met activation, which was blocked by knocking down CD74 expression using siRNA. MIF also induced Akt and associated FOXO3a phosphorylation, and this effect was abolished by knocking down either CD74 or Akt. In addition, MIF decreased oxidative stress in MSCs, as shown by decreased ROS and MDA, and increased the activity of SOD. Knockdown of CD74, Akt, or FOXO3a largely attenuated the anti-apoptotic effect of MIF and its ability to protect against oxidative stress. MIF protected MSCs from hypoxia/SD-induced apoptosis by interacting with CD74 to stimulate c-Met, leading to downstream PI3K/Akt-FOXO3a signaling and decreased oxidative stress.

## Introduction

In the absence of effective endogenous repair mechanisms after cardiac injury, cell-based therapies have rapidly emerged as a potential novel therapeutic approach in ischemic heart disease [1]. Allogeneic mesenchymal stem cells (MSC) are immunoprivileged, easily obtained, and improved heart function in preclinical and clinical studies, so have been widely used in the treatment of ischemic heart diseases [2]. Despite promising results, many problems, including massive cell death after transplantation, have limited the efficacy of this cell therapy [3]. At this stage, further optimization of MSC-based therapy is urgently needed, and growth factors and cytokines, preconditioning, and genetic modification, have all been manipulated in an attempt to enhance MSC survival [4–6]. Devising a more effective and accessible pro-survival strategy may make this therapeutic approach more attractive to the specialist.

Macrophage migration inhibitory factor (MIF) is a pleiotropic cytokine expressed in several cell types, including monocytes/macrophages, vascular smooth muscle, and cardiomyocytes [7–9], and serves as a regulatory factor in inflammation, apoptosis, autophagy, and carbohydrate metabolism [10–13]. Cell survival and proliferation is induced by MIF under stress conditions such as inflammation and starvation [11, 14–16]. With respect to apoptosis, MIF has been shown to act as an anti-apoptotic agent in various cells [10, 17], and recent research has confirmed that it can protect neural stem/progenitor cells from apoptosis [10]. Therefore, it is possible that MIF may be involved in a protective capacity against hypoxia/SD-induced MSC apoptosis.

CD74 is a well-known receptor for MIF. MIF binds to the CD74 extracellular domain to activate signaling pathways which play an anti-apoptotic role in some cells [18]. The phosphoinositide-3-kinase (PI3K)/Akt signaling pathway, which can be activated by MIF binding to CD74, functions in the cellular response to growth factors, and regulates key cellular functions including growth, metabolism, migration, apoptosis, and survival [17, 19, 20]. Forkhead transcription factor FOXO3a, the PI3K/Akt downstream substrate, is a positive regulator of cell survival in response to a variety of stress stimuli such as oxidative stress, DNA damage, and nutrient deprivation [21, 22], and activation of FOXO3a signaling has been shown to produce an anti-apoptotic effect in haematopoietic stem cells [19]. It has also been demonstrated that MIF stimulates the PI3K/Akt-FOXO3a signaling pathway [20], and that the MIF receptor CD74 is expressed on MSCs [23], in which the related PI3K/Akt-FOXO3a pathway exerts a protective effect [24, 25]. Thus, binding of MIF to CD74 and activation of the PI3K/Akt-FOXO3a pathway may facilitate resistance to hypoxia/SD-induced apoptosis in MSCs.

Reactive oxygen species (ROS) also play an important role in apoptosis induction under both physiologic and pathologic conditions. A previous study showed that ROS hinder adhesion of implanted MSCs to ischemic myocardium, resulting in apoptosis [26]. MIF not only functions primarily as a proinflammatory cytokine but also exhibits anti-oxidant properties by virtue of its intrinsic oxidoreductase activity [27]. Previous studies have confirmed that MIF protects cells from apoptosis by inhibiting ROS generation [28] and it is possible that MIF may protect MSCs from hypoxia/SD-induced apoptosis via a similar mechanism.

We hypothesized that exogenous MIF may prevent hypoxia/SD-induced apoptosis of MSCs and promote subsequent survival of these cells. The present study examined the effect of MIF on hypoxia/SD-induced apoptosis of MSCs and its related signaling pathways.

## Materials and methods

### Animals

Male Sprague–Dawley rats weighing 60–80 g were cared for in accordance with published guidelines from the U.S. National Institutes of Health. All study procedures were approved by the Harbin Medical University Institutional Animal Care and Use Committee. The study was conducted in compliance with the Guide for the Care and Use of Laboratory Animals published by the National Academy Press (NIH, revised 1996).

### Reagents

Dulbecco’s modified Eagle’s medium (DMEM) and fetal bovine serum (FBS) were obtained from Hyclone (Hyclone, Logan, UT).Trizol reagent was obtained from Invitrogen (Carlsbad, CA). The transcriptor First Strand cDNA Synthesis Kit, X-treme GENE HP DNA transfection reagent, and Fast Start Universal SYBR Master (ROX) were obtained from Roche (Mannheim, Germany). The annexin V-FITC Apoptosis Detection Kit, anti-CD44, anti-CD29, and anti-CD90 antibodies were obtained from BD Pharmingen (Franklin Lakes, NJ, USA). Anti-CD34 and anti-CD45 antibodies were obtained from eBioscience (San Diego, CA). Rabbit monoclonal antibodies against Akt, phospho-Akt (Ser473), phospho-Akt (Tyr308), phospho-FoxO1 (Thr24)/FoxO3a (Thr32), and FoxO3a were purchased from Cell Signaling Technology (Danvers, MA, USA). Rabbit monoclonal antibody CD74 was obtained from Santa Cruz Biotechnology (Santa Cruz, CA). Mouse polyclonal β-actin antibody was purchased from Zhongshan Goldenbridge Biotechnology (Zhongshan Goldenbridge Biotechnology Co. Ltd., #TA-09). Horseradish peroxidase-conjugated secondary antibodies to mouse or rabbit were obtained from Santa Cruz Biotechnology. Alexa Fluor 555 goat anti-rabbit IgG was obtained from Invitrogen. MIF ELISA kit was obtained from Rapidbio (CA, USA). Small interfering RNA (siRNA) to Akt and FOXO3a genes was obtained from Life Technologies (Carlsbad, CA) and siRNA to CD74 was obtained from QiaGen. Rat recombinant MIF was obtained from Prospec (NJ, USA). 2′,7′-dichlorodihydrofluorescein diacetate (DCF-DA) was obtained from Beyotine Institute of Biotechnology (Nantong, China). Rabbit monoclonal antibody c-Met, lipid peroxidation (MDA) assay kit, and SOD activity colorimetric assay kit were obtained from Abcam (Cambridge, UK). 3-(4,5,-Dimethyl thiazolyl-2)-2,5-diphenyl tetrazolium bromide(MTT) and dimethyl sulfoxide (DMSO) were purchased from Sigma-Aldrich (St Louis, MO, USA).

### Cell culture and treatment

Bone marrow-derived mesenchymal stem cells (BM-MSCs) were isolated from femurs and tibias of Sprague–Dawley rats as described previously [29]. Briefly, bone marrow cells were flushed from femurs and tibias with 5 mL DMEM/F12. After red blood cells were lysed and removed, 5 × 10^5^ cells were plated in a 25-cm^2^ flask with 6 mL DMEM/F12 supplemented with 10 % FBS and 1 % penicillin/streptomycin at 37 °C with 5 % CO_2_. After 3 days in culture, non-adherent cells were washed off with medium and adherent MSCs were further grown in medium replaced every 3 days. At 80–90 % confluence, adherent cells were trypsinized, diluted 1:2–2:3, and re-expanded. All cells used in each assay were at passages 3–5. The characteristics of MSCs were demonstrated by immunophenotyping as described previously [29].

For MIF stimulation, cells were refreshed with medium containing 100 ng/mL of recombinant MIF and incubated at 37 °C for various periods as previously reported [17]. Induction of apoptosis in vitro by hypoxia and serum deprivation (hypoxia/SD), designed to mimic the in vivo conditions of ischemic myocardium, was initiated as previously reported [30]. Apoptosis was induced by incubating MSCs in serum-free media in a controlled atmosphere (anaerobic chamber) glove box (Plas Labs 855-AC) to scavenge free oxygen. MIF (100 ng/mL) was added to the medium at the beginning of hypoxia/SD exposure and incubation was continued for 24 h under hypoxic conditions. Cells exposed to hypoxia/SD alone without MIF exposure were used as apoptotic controls.

### Flow cytometric analysis of cell apoptosis

Apoptosis was determined by detecting phosphatidylserine exposure on cell plasma membranes with the fluorescent dye Annexin V-FITC Apoptosis Detection Kit, according to manufacturer’s protocol. This assay discriminates between intact (Annexin V^−^/propidium iodide (PI)^−^, early apoptotic (Annexin V^+^/PI^−^), late apoptotic (Annexin V^+^/PI^+^), and necrotic (Annexin V^−^/PI^+^) cells. Briefly, cells were harvested and washed in ice-cold PBS, resuspended in 300 µL binding buffer, and incubated with 5 µL Annexin V-FITC solution for 30 min at 4 °C in dark conditions. This was followed by a further incubation in 5 µL PI for 5 min and immediate analysis of the cells by bivariate flow cytometry using the BD FACSCanto II equipped with BD FACSDiva Software (Becton–Dickinson, San Jose, CA). Approximately 1–5 × 10^5^ cells were analyzed in each sample.

### MTT assay

We tested the potential toxicity of MIF in cultured MSCs. The cells were incubated with increasing concentrations of MIF in culture medium for 24 h. A total of 300 µL of MTT reagent were added to each well 2 h prior to harvesting. The supernatant was then removed and incubated with 400 µL of DMSO for 10 min. Absorbance at 540 nm was recorded using an enzyme-linked immunosorbent assay plate reader.

### ELISA

Secretion of MIF was measured in cell culture media using an ELISA kit. The assays were conducted in 96-well microplates according to the manufacturer’s instructions.

### Small interfering RNA (siRNA) knockdown

Cells were transfected using X-treme GENE HP DNA Transfection Reagent, according to the manufacturer’s instructions. In brief, MSCs were plated in a 6-well plate and treated with X-treme GENE HP DNA Transfection Reagent in a 3:1 ratio of reagent volume (μL) to siRNA mass (μg) for 20 min. Cells were then transfected with a mixture containing 100 nM siRNA and incubated in 2 mL of culture medium for 48 h. Scrambled siRNA (siRNA-NT) was used as the control. Transfection efficiency of siRNA-CD74, siRNA-Akt, and siRNA-FOXO3a was analyzed by Western blotting of the relevant proteins compared to scrambled siRNA controls.

### Real time polymerase chain reaction

Gene expression was analyzed by quantitative real time polymerase chain reaction (qRT-PCR). Briefly, total RNA was isolated and reverse transcribed to cDNA using the transcriptor First Strand cDNA Synthesis Kit according to the manufacturer’s instructions. qPCR was carried out with the Fast Start Universal SYBR Master and fluorescence quantitative PCR system. The threshold cycle was set within the exponential phase of the PCR. Relative gene expression was calculated by comparing cycle times for each target PCR. The target PCR Ct values were normalized by subtracting the GAPDH Ct value, which provided the △Ct value. The relative expression level between treatments was then calculated using the following equation: relative gene expression = 2^−(△Ct sample–△Ct control)^.

### Western blotting analysis

Western blotting experiments were carried out as previously described [29]. Briefly, cells were washed twice with ice-cold PBS and ruptured with lysis buffer containing 20 mM Tris–HCl, 150 mM NaCl, 1 % Triton X-100, and protease and phosphatase inhibitors. Cell extracts were centrifuged for 5 min at 12,000×*g* and supernatants were collected. In all experiments, 20 µg of cellular protein was resolved by SDS-PAGE and transferred onto PVDF membranes. Membranes were blocked for 1 h with 5 % skim milk in Tris-buffered saline containing 0.1 % Tween 20 and incubated overnight at 4 °C with primary antibodies. Membranes were washed, incubated for 1 h with appropriate secondary antibodies conjugated to horseradish peroxidase, and developed using chemiluminescence substrates, photographed with BIO-RAD ChemiDoc XRS equipment, quantified, and analyzed with Quantity One software.

### Immunofluorescence staining

To investigate the expression of CD74 on the surface of MSCs, cells were grown on glass coverslips and fixed with 4 % paraformaldehyde for 15 min at room temperature, blocked with 10 % BSA and incubated with anti-CD74 antibody at 4 °C over night. After washing, the cells were incubated with the Alexa Fluor 555-conjugated goat anti-rabbit IgG for 1 h at 37 °C. The nuclei of cells were counterstained with 4′,6-diamidino-2-phenylindole. Fluorescence images were acquired with a fluorescence microscope.

### ROS measurement

The level of intracellular ROS was determined using 2,7-dichlorodihydrofluorescein diacetate (DCFH-DA), following the manufacturer’s instructions. The fluorescence intensity of cells was measured with a fluorescence spectrophotometer, with excitation and emission wavelengths of 488 and 525 nm, respectively.

### SOD activity

SOD activity was determined in MSCs using a colorimetric assay kit (Abcam) according to the manufacturer’s protocol. Briefly, protein was isolated using lysis buffer from control and MIF-exposed MSCs and SOD activity was measured using 10 ug of the total protein extract. Absorbance values were measured at 450 nm.

### Lipid peroxidation assays

Lipid peroxidation was monitored using an assay kit (Abcam) to measure formation of MDA according to the manufacturer’s protocol. Briefly, MSCs (1 × 10^6^ cells) were homogenized on ice in 300 μl of MDA Lysis Buffer (with 3 μl BHT (100×), then centrifuged (13,000×*g*, 10 min) to remove insoluble material. Supernatant (200 μl) was added to 600 μl of thiobarbituric acid and incubated at 95 °C for 60 min. The samples were cooled to room temperature in an ice bath for 10 min, and absorbance at 532 nm was measured spectrophotometrically.

### Statistical analysis

Data were expressed as mean ± SD. Differences among groups were tested by one-way ANOVA, and comparisons between two groups were evaluated using Student’s *t* test provided by the statistical software SPSS package v19.0 (SPSS, Inc., Chicago, IL, USA). A value of *P* < 0.05 was considered statistically significant.

## Results

### MSCs produced MIF upon exposure to hypoxia/SD

**Fig. 1:**
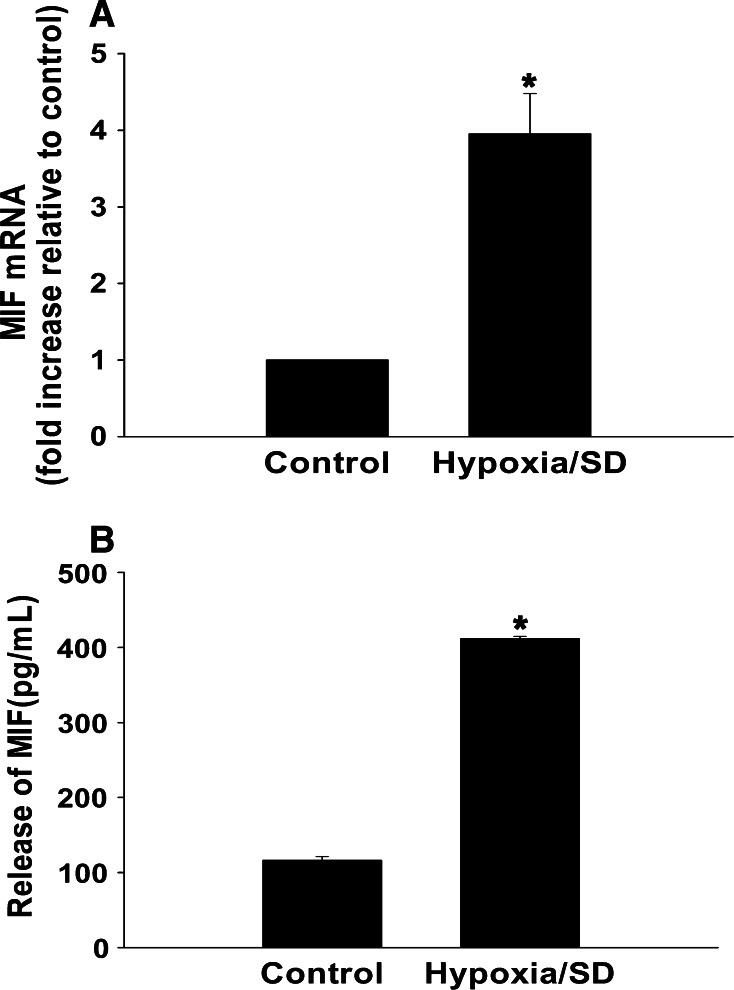
MSCs produced MIF upon exposure to hypoxia/SD. MSCs were cultured under hypoxic/SD or normal conditions for 24 h. (**a**) MIF mRNA levels were analyzed by qRT-PCR as described in the methods. (**b**) Release of MIF was analyzed by ELISA. Each column represents the mean ± SD of three independent experiments; *P < 0.05 versus control

As a first step, we examined MIF mRNA expression under hypoxia/SD using qRT-PCR and found that MIF mRNA level increased approximately fourfold compared with the normoxic control (Fig. 1a). Moreover, we found that hypoxia/SD stimulated MIF release. There was an approximately fourfold increase in release of MIF into the medium as compared to control cells in normoxia (Fig. 1b).

### MIF protected MSCs from hypoxia/SD-induced apoptosis

**Fig. 2:**
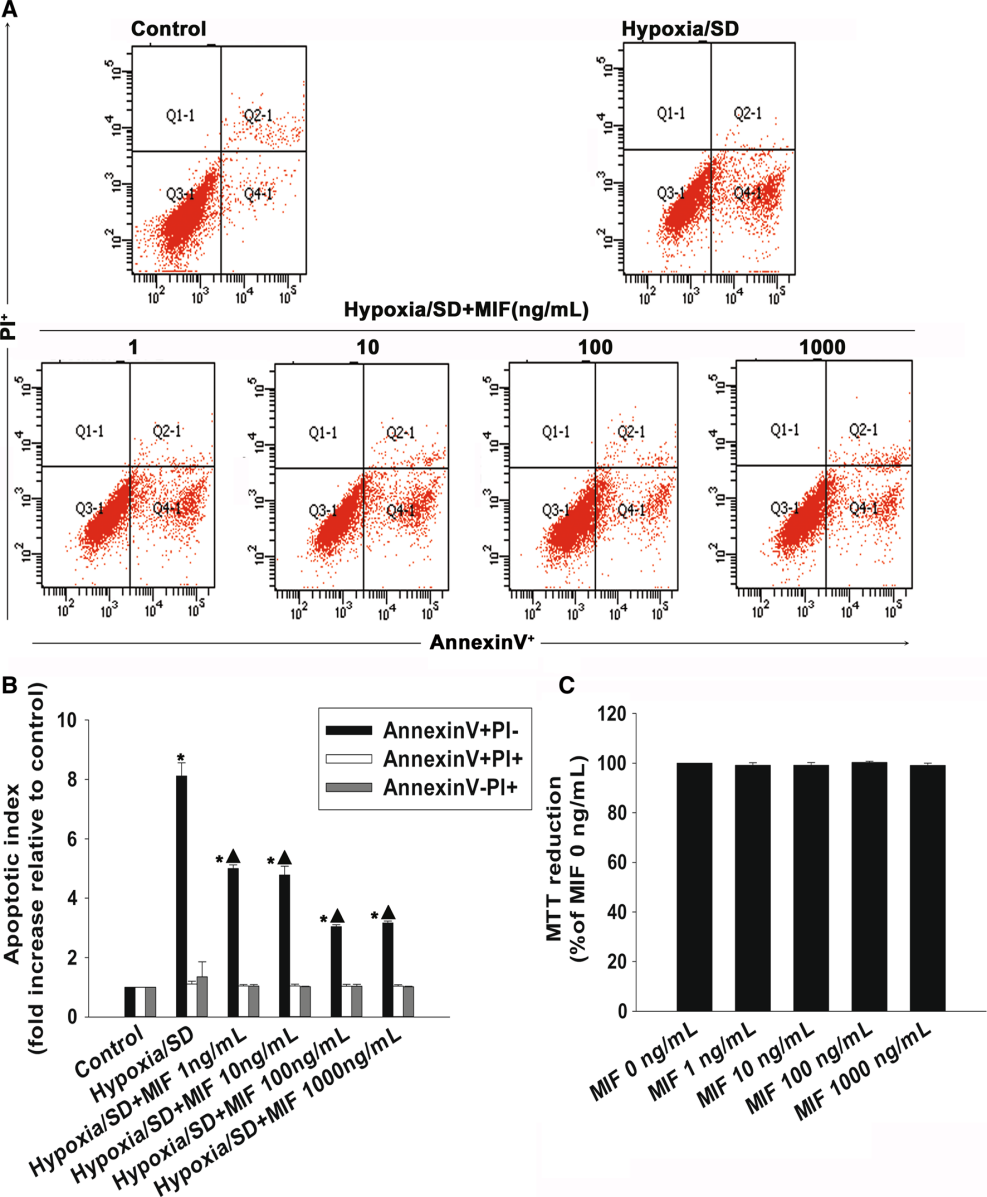
MIF protected MSCs against hypoxia/SD-induced apoptosis. MIF (1–1000 ng/mL) was added at the point of exposure to hypoxia/SD and was maintained in the incubation medium throughout the hypoxia/SD treatment period. Apoptosis was measured by flow cytometry. (**a**) Representative of three FACS can flow cytometric analyses of apoptotic cells after Annexin V and PI staining. (**b**) Fold-changes compared with corresponding control cells. Each column represents the mean ± SD of three independent experiments. *P < 0.05 versus control, *Filled triangle* *P* < 0.05 versus hypoxia/SD. (**c**) MIF was added to culture media alone in order to test its effect on MSC viability, and analyzed for their viability by MTT assay. The MTT assay indicated that MIF up to 1000 ng/mL was not toxic to cultured MSCs. Each column represents the mean ± SD of three independent experiments

In preliminary experiments, hypoxia/SD induced MSC apoptosis with a maximal induction of early apoptosis at 24 h [29]. We then asked if MIF could protect MSCs from hypoxia/SD-induced apoptosis. MSCs were exposed to various concentrations of MIF (1–1000 ng/mL) followed by exposure to hypoxia/SD for 24 h. Cell apoptosis was determined by FACS. The data showed a clear anti-apoptotic effect of MIF (Fig. 2) as MIF at any concentration efficiently blocked apoptosis. The extent to which cells entered apoptosis was examined by monitoring staining with Annexin V-FITC at the end of the incubation period (Fig. 2a, b). Results showed that MIF significantly inhibited hypoxia/SD-induced apoptosis, with the most pronounced effects observed at 100–1000 ng/mL (100 ng/mL: 3.04 ± 0.063 vs. 8.12 ± 0.44, *P* < 0.05; 1000 ng/mL: 3.17 ± 0.063 vs. 8.12 ± 0.44, *P* < 0.05).

To assure that MIF was not toxic to MSCs at these concentrations, we examined toxic effects on cell viability using an MTT assay. MIF had no adverse effect on the viability of MSCs at concentrations up to 1000 ng/mL (Fig. 2c).

### MIF protected MSCs from apoptosis through a MIF-CD74-dependent molecular pathway

**Fig. 3:**
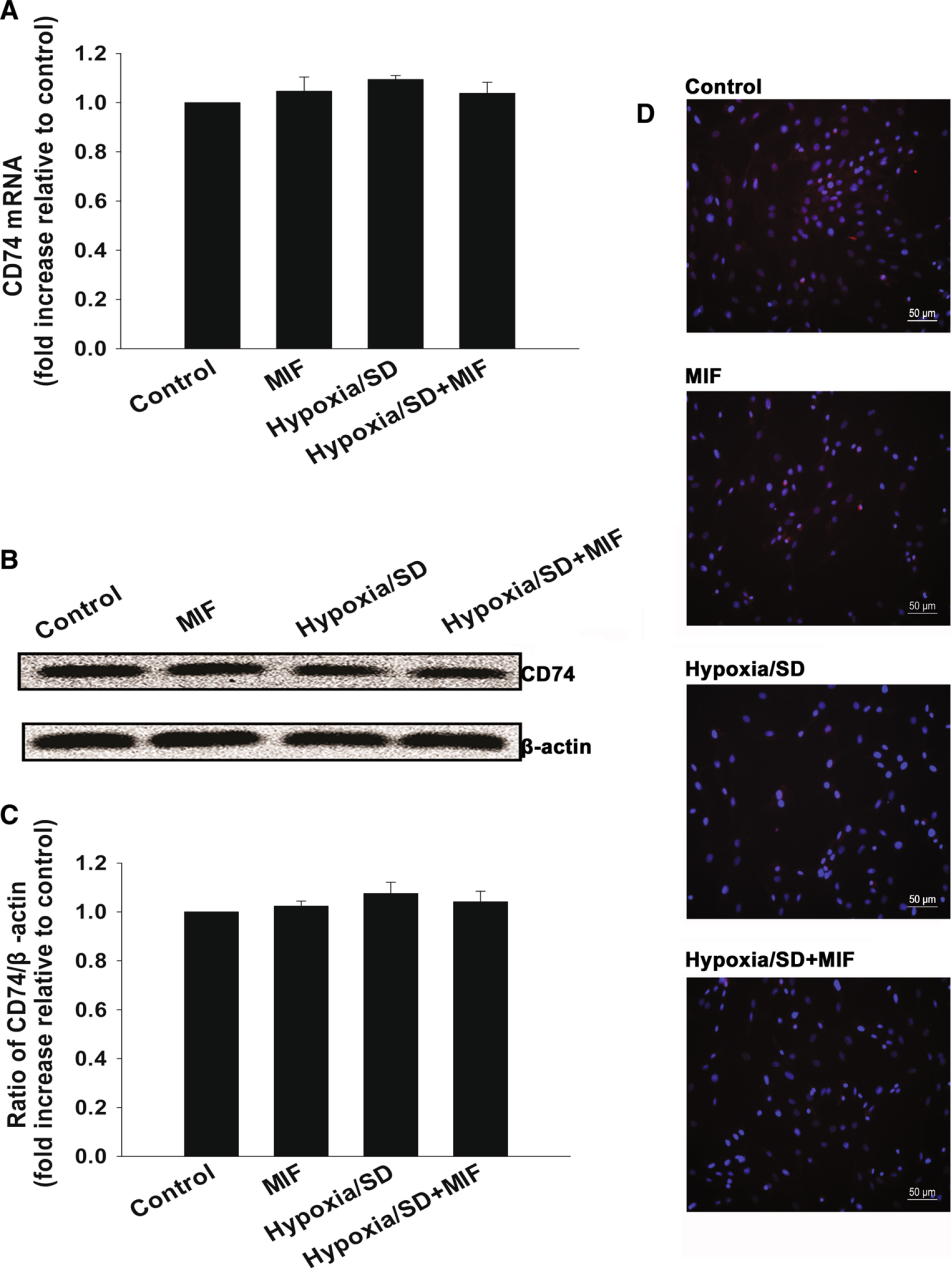
MIF influenced expression of CD74 in MSCs. (**a**, **b**) Expression of CD74 mRNA (**a**) analyzed by qRT-PCR, and protein (**b**) analyzed by Western blot. Each column represents Mean ± SD from three independent experiments; *P* > 0.05. (**c**) Densitometric quantification of CD74 expression relative to internal control β-actin. (**d**) Expression of CD74 on the cell surface of MSCs was analyzed by immunofluorescence assay

Because CD74 is an important receptor for MIF, we tested if MIF protected MSCs from apoptosis via an interaction with CD74 by evaluating CD74 expression in hypoxia/SD-treated MSCs. As shown in Fig. 3a–c, the mRNA and protein levels of CD74 were the same whether treated with hypoxia/SD or MIF. We also verified expression of CD74 by immunofluorescent staining (Fig. 3d).

**Fig. 4:**
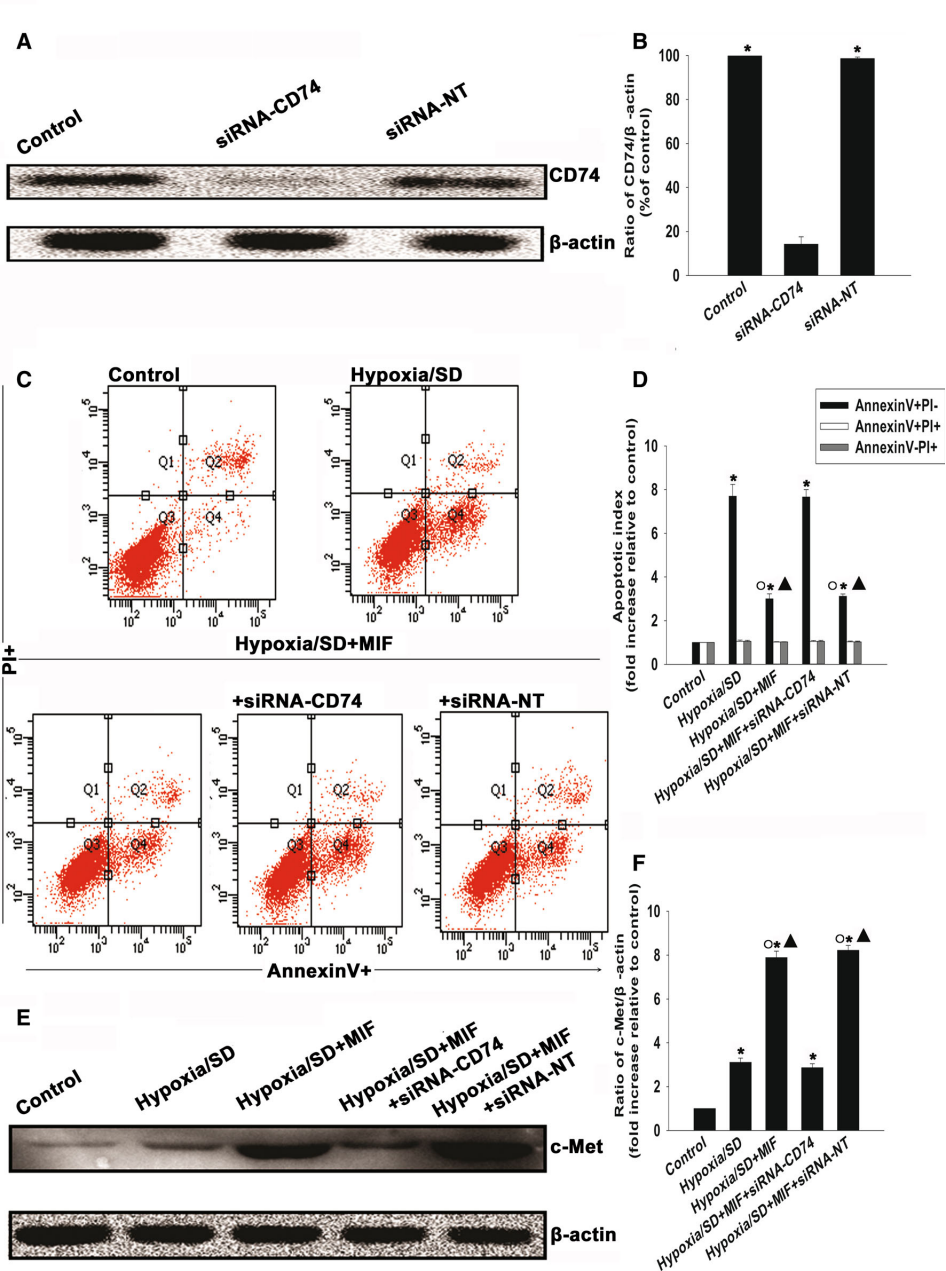
MIF protected MSCs from apoptosis through a MIF/CD74-related molecular pathway. (**a**, **b**) MSCs were transfected with siRNA against the CD74 transcript or with siRNA-NT as control. siRNA-mediated transfection efficiency was determined by Western blot. Each column represents the mean ± SD of three independent experiments; *P < 0.05 versus siRNA-CD74. (**c**, **d**) MSCs were incubated under hypoxic/SD conditions for 24 h. In parallel experiments, cells were treated with MIF (100 ng/mL) in the incubation medium throughout the hypoxia/SD treatment period. Apoptosis was analyzed by FACS. Each column represents the mean ± SD of three independent experiments; *P < 0.05 versus control; *Filled triangle* *P* < 0.05 versus hypoxia/SD, *White circle P* < 0.05 versus hypoxia/SD + siRNA-CD74. (**e**, **f**) Expression levels of c-Met were analyzed by Western blot assay. Each column represents the mean ± SD of three independent experiments; *P < 0.05 versus control; *Filled triangle* *P* < 0.05 versus hypoxia/SD, *White circle* *P* < 0.05 versus hypoxia/SD + siRNA-CD74

To further confirm the role of CD74 in the MIF anti-apoptotic effect, we inhibited CD74 expression using siRNA and examined apoptosis of MSCs under hypoxic/SD conditions. As shown in Fig. 4a, b, siRNA transfection significantly decreased the expression of CD74 compared to control (14.22 ± 3.35 vs. 100.00 ± 0.00 %, *P* < 0.05). Silencing CD74 significantly attenuated the anti-apoptotic effect of MIF (7.67 ± 0.34 vs. 3.00 ± 0.23, *P* < 0.05). In contrast, no difference was found when MSCs were transfected with siRNA-NT (3.11 ± 0.11 vs. 3.00 ± 0.23; Fig. 4c, d).

Because CD74 is known to activate cellular c-Met, we investigated c-Met expression in MSCs following MIF exposure. As shown in Fig. 4e, f, MIF treatment increased expression of c-Met (7.89 ± 0.29 vs. 1.00 ± 0.00, *P* < 0.05) compared to the control. Transfection of MSCs with siRNA-CD74 abolished the effect of MIF (2.86 ± 0.19 vs. 7.89 ± 0.29, *P* < 0.05; Figs. 4e, f).

### MIF protected MSCs from hypoxia/SD-induced apoptosis through CD74-sensitive PI3K/Akt-FOXO3a pathways

**Fig. 5:**
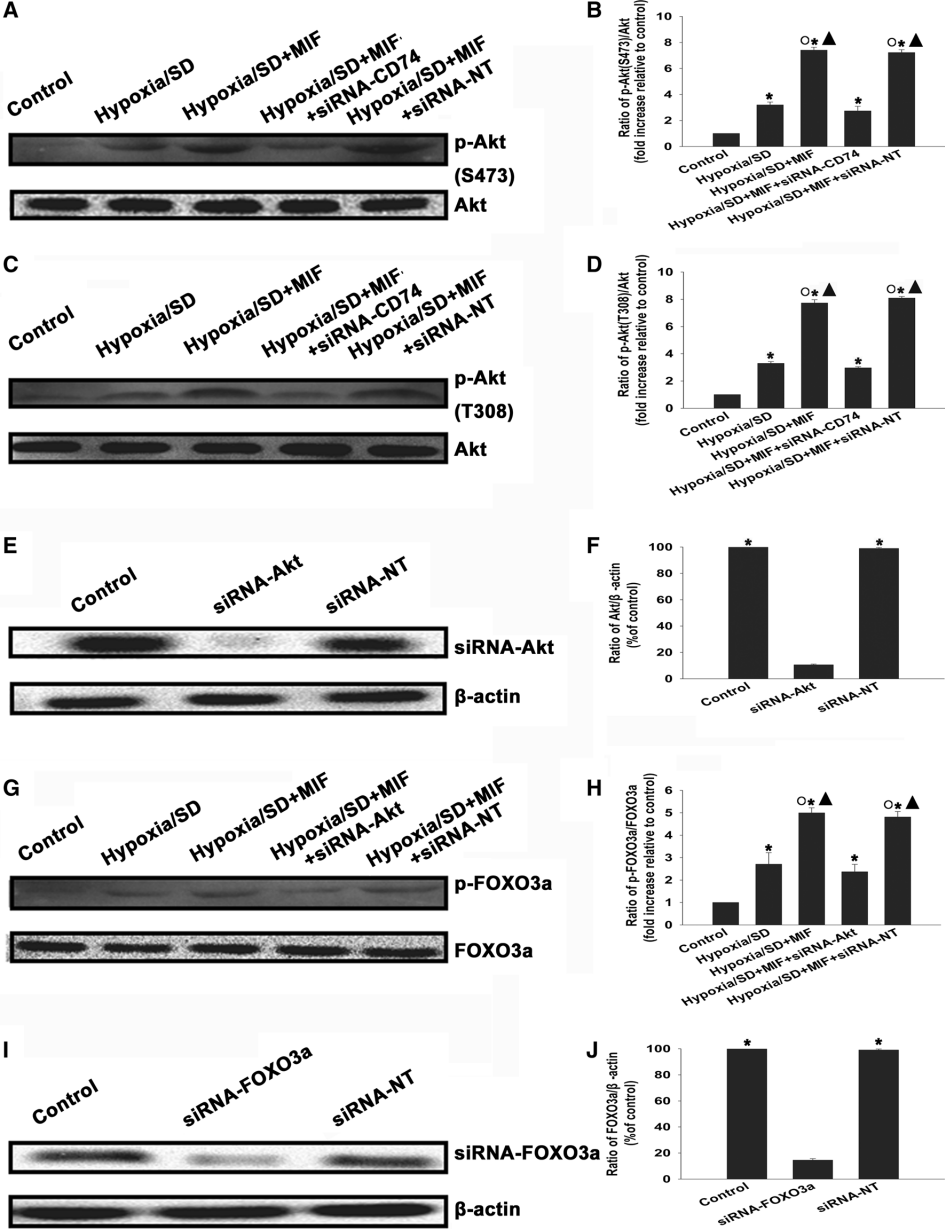
MIF-induced activation of the PI3K/Akt-FOXO3a pathway. (**a**–**d**) Representative images of Western blots showing Akt and phospho-Akt expression. Cells were transfected with siRNA-CD74 or siRNA-NT before treatment with MIF (100 ng/mL) and incubation in hypoxic/SD conditions for 24 h. Fold change in phospho-Akt expression was compared to Akt. Each column represents the mean ± SD of three independent experiments; *P < 0.05 versus control; *Filled triangle* *P* < 0.05 versus hypoxia/SD, *White circle* *P* < 0.05 versus hypoxia/SD +siRNA-CD74. (**e**, **f**) MSCs were transfected with siRNA against Akt or with siRNA-NT. The siRNA-mediated transfection efficiency was determined by Western blotting. Each column represents the mean ± SD of three independent experiments; *P < 0.05 versus siRNA-Akt). (**g**, **h**) Representative images of Western blots of FOXO3a and phospho-FOXO3a. Cells were transfected with siRNA-Akt or siRNA-NT before treatment with MIF (100 ng/mL) and incubation under hypoxic/SD conditions for the indicated time. Fold change in phospho-FOXO3a was compared to FOXO3a. Each column represents the mean ± SD of three independent experiments; *P < 0.05 versus control; *Filled triangle* *P* < 0.05 versus hypoxia/SD, *White circle P* < 0.05 versus hypoxia/SD + siRNA-Akt. (**i**, **j**) MSCs were transfected with siRNA against FOXO3a or with siRNA-NT. siRNA-mediated transfection efficiency was determined by Western blotting. Each column represents the mean ± SD of three independent experiments; *P < 0.05 versus siRNA-FOXO3a

The PI3K/Akt signaling pathway is an important regulator promoting survival of many cell types. Therefore, we investigated whether this pathway mediated the anti-apoptotic effect of MIF in MSCs. Western blotting analysis showed low detectable levels of phospho-Akt in control cells. MIF induced a pronounced increase in Akt phosphorylation at two sites (S473: 7.41 ± 0.23 vs. 1.00 ± 0.00, *P* < 0.05; T308: 7.73 ± 0.24 vs. 1.00 ± 0.00, *P* < 0.05) which was abolished by silencing CD74 expression (S473: 2.74 ± 0.35 vs. 7.41 ± 0.23, *P* < 0.05; T308: 2.87 ± 0.08 vs. 7.73 ± 0.24, *P* < 0.05; Fig. 5a–d). Downstream from PI3K/Akt, we found that MIF also induced significant phosphorylation of FOXO3a (5.00 ± 0.23 vs. 1.00 ± 0.00, *P* < 0.05). Silencing Akt expression with siRNA-Akt (Fig. 5e, f) abolished the effect of MIF on FOXO3a phosphorylation (2.37 ± 0.33 vs. 5.00 ± 0.23, *P* < 0.05; Fig. 5g, h).

**Fig. 6:**
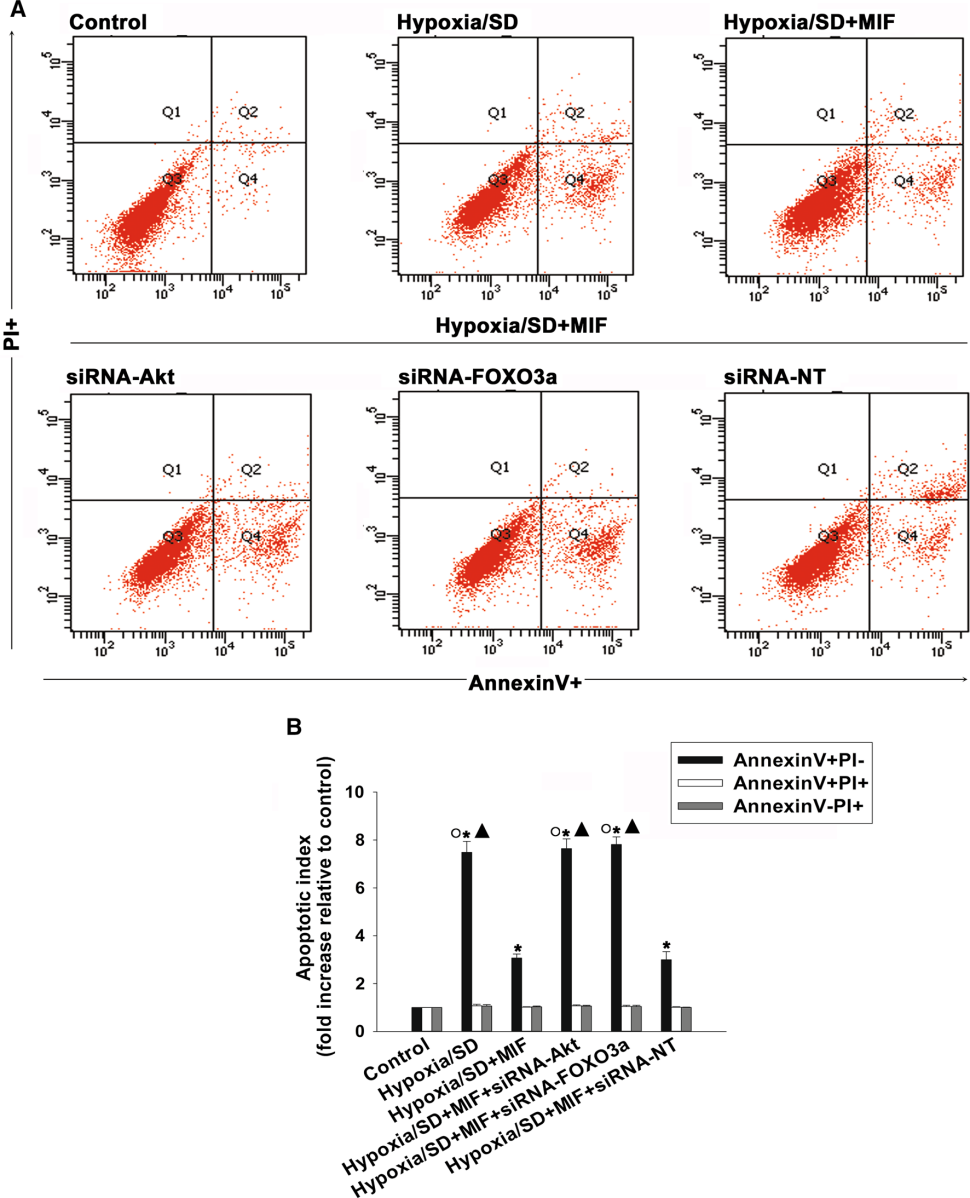
MIF protected MSCs from hypoxia/SD-induced apoptosis through a CD74-sensitive PI3K/Akt-FOXO3a pathway. (**a**, **b**) To determine which kinase pathway was involved in the anti-apoptotic actions of MIF, MSCs were transfected with siRNA against Akt or FOXO3a, or with siRNA-NT as a control. MSCs were then incubated under hypoxic/SD conditions for 24 h. In parallel experiments, cells were treated with MIF (100 ng/mL) during exposure to hypoxia/SD. The apoptosis rate was analyzed by flow cytometry. Each column represents the mean ± SD of three independent experiments; *P < 0.05 versus control; *Filled triangle* *P* < 0.05 versus hypoxia/SD + MIF, *White circle P* < 0.05 versus hypoxia/SD + siRNA-NT

To further confirm the role of PI3K/Akt and FOXO3a in the anti-apoptotic effect of MIF, we silenced Akt and FOXO3a using siRNA and examined apoptosis of MSCs under hypoxic/SD conditions. As shown in Fig. 5, knockdown of Akt or FOXO3a gene expression by siRNA decreased the level of Akt and FOXO3a protein to 10.59 ± 0.55 and 14.51 ± 1.13 %, respectively, compared to control cells. Silencing of either Akt or FOXO3a significantly attenuated the anti-apoptotic effect of MIF (siRNA-Akt: 7.64 ± 0.41 vs. 3.07 ± 0.17, *P* < 0.05; siRNA-FOXO3a: 7.81 ± 0.32 vs. 3.07 ± 0.17, *P* < 0.05; Fig. 6a, b). In contrast, transfection with siRNA-NT did not alter the anti-apoptotic effect of MIF (3.00 ± 0.34 vs. 3.07 ± 0.17).

### MIF showed anti-apoptotic effects via inhibition of oxidative stress

**Fig. 7:**
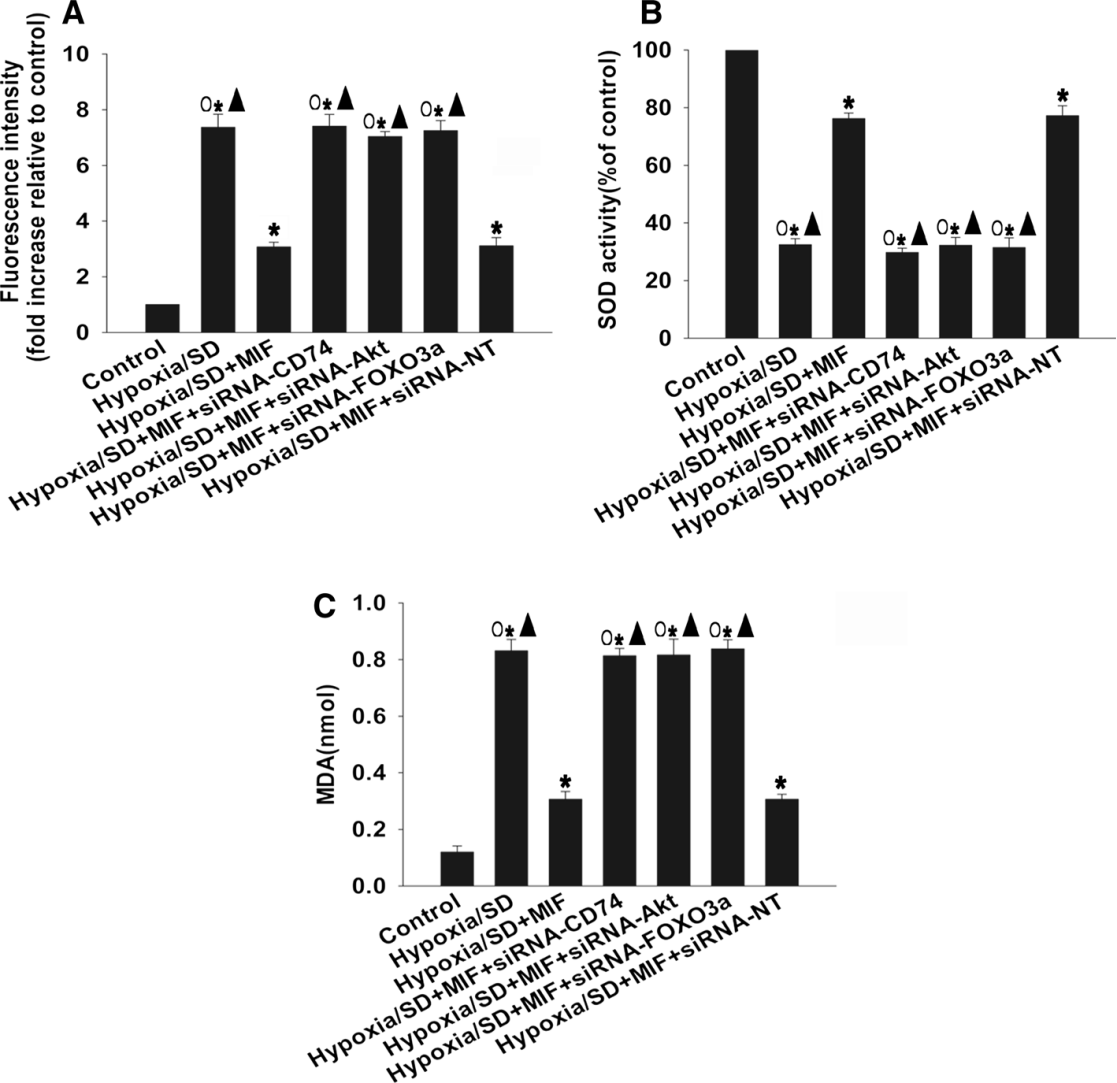
MIF exerted anti-apoptotic effects via inhibition of oxidative stress. MSCs were incubated under hypoxic/SD conditions for 24 h. In parallel experiments, cells were transfected with siRNA-CD74, siRNA-Akt, or siRNA-FOXO3a before exposure to hypoxia/SD. When used, MIF (100 ng/mL) was added at the beginning of exposure to hypoxia/SD. (**a**) Intracellular ROS production in MSCs was analyzed by fluorescence spectrophotometer. (**b**) SOD activity was evaluated using a colorimetric assay. (**c**) MDA formation was used to evaluate lipid peroxidation. Each column represents the mean ± SD of three independent experiments; *P < 0.05 versus control; *Filled triangle* *P* < 0.05 versus hypoxia/SD + MIF, *White circle P* < 0.05 versus hypoxia/SD + MIF + siRNA-NT

To determine whether the anti-apoptotic effects of MIF involved oxidative stress under hypoxic/SD conditions, we examined generation of ROS, activation of SOD, and lipid peroxidation by MDA assay. MIF increased activation of SOD (76.22 ± 1.93 vs. 32.48 ± 2.04 %, *P* < 0.05), decreased generation of ROS (3.07 ± 0.17 vs. 7.37 ± 0.47, *P* < 0.05), and reduced MDA activation (0.31 ± 0.03 vs. 0.83 ± 0.04, *P* < 0.05; Fig. 7). siRNAs against CD74, Akt, or FOXO3a were all potent blockers of MIF inhibition (Figs. 7a–c).

## Discussion

Autologous MSCs offer a great advantage when used to regenerate and repopulate injured myocardium, restoring heart function after transplantation into ischemic or infarcted heart. Autologous MSCs can be easily prepared from adult patients and they are immunologically safe [31]. However, the ratio of surviving cells following MSC engraftment is extremely low despite the large number of implanted cells, probably due to a high rate of cell death induced by the ischemic environment [5, 32], where transplanted MSCs encounter nutrient and oxygen deprivation and oxidative stress. In such a stressful environment, it is likely that many transplanted cells undergo apoptosis, so enhancing the survival of MSCs is a significant challenge for stem cell-based therapy [24]. In the present study, we show that the anti-oxidant effect induced by MIF protects MSCs from hypoxia/SD-induced apoptosis via a CD74-dependent Akt-FOXO3a-related pathway, likely by enhancing MSC survival in the face of growth factor fluctuations and nutrient deprivations that naturally occur in the ischemic microenvironment, especially after cardiac infarction.

MIF is an important cytokine mediating anti-oxidant and stress responses [33]. MIF has diverse enzymatic activities which reflect its various functions in cells, including participation in cell survival, proliferation, and migration [34–36]. MIF is overexpressed in various stress conditions such as ischemia, hypoxia, and oxidation, and it exerts an apparent cellular protective effect through repair of DNA damage, modulation of p53 gene expression, and reduction in oxidative stress [37, 38].With regard to apoptosis, MIF is also known to affect signaling in growth and apoptosis-regulating pathways, and is a powerful anti-apoptotic agent in various cells [39, 40]. In our investigation, culture of MSCs under hypoxic/SD conditions-induced MIF generation, in agreement with previous studies showing that cellular stress-induced MIF overexpression [9]. In those studies, insufficient MIF was generated to protect MSCs from hypoxia/SD-induced apoptosis, but our results showed that exogenously increasing MIF protected MSCs from hypoxia/SD-induced apoptosis in a concentration-dependent manner.

CD74, a type II transmembrane glycoprotein that acts as an MHC class II chaperone, was previously described as a cognate ligand for MIF [18]. Subsequent experiments demonstrated that MIF initiated a signaling cascade which resulted in cell survival in a CD74-dependent manner [10, 17, 20]. In our experiments, hypoxia/SD or MIF failed to alter expression of CD74, but using siRNA to silence CD74 expression significantly attenuated the anti-apoptotic effect of MIF, confirming that MIF exerted its anti-apoptotic effect in a CD74-dependent manner. CD74 does not have a typical signal-transducing cytosolic domain, but may utilize a signal-transducing coreceptor pathway in which recruitment of CD44 activates non-receptor tyrosine kinases [18] A previous study demonstrated that stimulation of CD74 by MIF recruits the tyrosine kinase receptor c-Met to the CD74/CD44 complex and thereby enables induction of a signaling cascade within the cell to promote B cell survival [41]. Activated c-Met triggers a cascade of signal transduction pathways including activation of PI3K and Akt, which have been implicated in cell migration, cell motility, cell cycle progression, and cell survival [42–44]. In our study, MIF-induced c-Met activation, which was inhibited by silencing CD74 expression, confirming that the anti-apoptotic effect of MIF was dependent on CD74/c-Met complex formation.

In addition to its role in the regulation of cell motility, cytoskeletal rearrangement and glucose metabolism, the PI3K/Akt pathway is best known for controlling survival through functional modulation of various gene products [45, 46]. Akt is a stress-signaling kinase and key regulator of energy generating and consuming pathways which protect cells against hypoxic injury and death. The PI3K/Akt signaling pathway has been shown to have a major anti-apoptotic effect in MSCs under hypoxia/SD conditions [5, 29], and MIF has been shown to activate this pathway [47]. Our results demonstrated that MIF activated CD74-dependent c-Met, and this was linked to activation of the Akt pathway and enhancement of cell survival by MIF. Silencing Akt attenuated the anti-apoptotic effect of MIF, suggesting that the protective effect of MIF was dependent on the PI3K/Akt signaling pathway.

FOXO factors are well known for their role in regulating stress responses. They can be activated by nutrient deprivation, oxidative stress, or DNA damage. In response to such a wide range of stress stimuli, FOXO factors regulate a broad variety of cellular mechanisms including DNA repair, scavenging of ROS, cell cycle progression, apoptosis, and metabolism [21, 48, 49]. As previously reported, in response to hypoxia FOXO3a transcript levels accumulated, suggesting that FOXO3a plays an important role in the survival response to hypoxic stress [21]. In addition, FOXO3a directed a protective autophagy program in haematopoietic stem cells [19]. In our study, we present evidence that MIF stimulated activation of Forkhead transcription factor FOXO3a to protect MSCs from hypoxia/SD-induced apoptosis and showed that this protection was attenuated by silencing of FOXO3a. Furthermore, the activity of FOXO3a induced by MIF during hypoxia/SD was also regulated by the PI3K/Akt pathway, because phospho-FOXO3a levels increased when treated with MIF, but decreased when Akt was silenced. This result agrees with a previous report showing that phospho-FOXO3a levels increased during hypoxia [21] and suggests that FOXO3a activity during hypoxia is regulated by the PI3K pathway.

With respect to apoptosis, oxidative stress is in general associated with induction of cell death [50]. Oxidative stress is accompanied by ROS generation, an increase in oxidant enzyme activity, and diminished anti-oxidant enzyme activity [51]. Here, we show that hypoxia/SD increased generation of ROS and MDA activity and decreased activation of SOD in MSCs, while treatment with MIF reversed these effects, confirming that MIF decreased oxidative stress, as shown by previous studies demonstrating that MIF was an effective anti-oxidant agent [28, 52]. Silencing of CD74, Akt, or FOXO3a diminished MIF inhibition of oxidative stress, suggesting that MIF exerts its anti-oxidative stress effects through a CD74-dependent Akt-FOXO3a signaling pathway.

In conclusion, we propose that MIF promotes MSC survival under conditions mimicking ischemic myocardium. The results of the present study suggest that MIF protects MSCs from hypoxia/SD-induced apoptosis via regulation of oxidative stress in a CD74-dependent Akt-FOXO3a signaling pathway. These findings highlight potential novel therapeutic strategies for protecting MSCs from apoptosis, and provide a mechanistic understanding for the clinical exploitation of MIF and MSCs in cardiac regeneration therapies.
